# Effects of alcohol consumption on driving performance in the presence of interocular differences simulated by filters

**DOI:** 10.1038/s41598-023-45057-8

**Published:** 2023-10-17

**Authors:** Francesco Martino, José J. Castro-Torres, Miriam Casares-López, Sonia Ortiz-Peregrina, Pilar Granados-Delgado, Rosario G. Anera

**Affiliations:** Laboratory of Vision Sciences and Applications (LabVisGra), Department of Optics, University of Granada, Granada, Spain

**Keywords:** Health care, Risk factors, Optics and photonics

## Abstract

The role of interocular differences simulated by filters (fog filter and Bangerter foil) on visual and driving performance in alcohol users was assessed. We found that the binocular visual function deteriorates significantly in terms of contrast sensitivity (from 6 to 18 cpd). Additionally, driving performance is significantly impaired under these conditions as evidenced by increased mean speed, standard deviation of the lateral position, distance traveled outside the lane, reaction time and number of collisions. Furthermore, we found that interocular differences due to intraocular scattering and straylight are directly related to an overall reduction in visual and driving performance. This provided a comprehensive perspective from which to understand the relationship between binocular visual function, interocular differences, and driving performance. In practice, our findings contribute to the understanding of the importance of limiting interocular differences, which can be common among presbyopes corrected using the monovision technique, as well as in cases of cataract or other ocular pathology affecting only one eye, or even in cases of cataract surgery of the first eye. These interocular differences can have an adverse impact on road safety, especially when combined with moderate alcohol consumption.

## Introduction

It is well established that alcohol is the most widely consumed psychoactive substance in the world^1^. Alcohol consumption is one of the leading causes of death in the world; according to the World Health Organization^1^, three million people died in 2016 because of alcohol, including almost 400,000 in alcohol-related traffic accidents. As a psychoactive substance, alcohol plays an important role as a central nervous system depressor^2^ that alters both driving ability and attitude, increasing the risk of being involved in a traffic accident. The greater the quantity of alcohol in the body, the greater the risk of an accident. For instance, a blood alcohol content (BAC) of 0.8 g of ethanol per liter of blood (g/l) increases the risk of an accident by five times (compared to no alcohol-use), and this risk continues to go up as the BAC rises^3–5^. In this regard, the most common legal limit worldwide for driving (in 51 countries including Spain) corresponds to a breath alcohol content (BrAC) of 0.25 mg/l (0.25 mg of ethanol per liter of exhaled air, equivalent to a BAC of 0.5 g/l or 0.05%). However, a BrAC of 0.40 mg/l (equivalent to a BAC of 0.8 g/l or 0.08%) is still the legal limit for driving in 45 countries around the world, including the UK and the USA^1,6^. People driving under the influence of alcohol (DUI) make many more mistakes at the steering wheel^7–9^ drive faster^7,8^, and experience worse decision making and increased reaction times^8,10–12^. Charlton and Starkey^13^ found dose-dependent alcohol impairment in driving and cognitive performance for two measured alcohol contents corresponding to BACs of 0.05% and 0.08%. In addition, alcohol produces significant behavioral alterations and affects the psychophysical capacities required for safe driving^14^. In particular, it significantly impairs binocular visual acuity^15,16^, contrast sensitivity^7^, and stereopsis^17,18^. The latter is defined as the capacity of the visual system to see the surrounding environment in depth. This binocular visual performance is influenced by several other factors including refractive error^19^, pupil size^20^, ocular pathologies^21,22^, and interocular differences^23,24^. An interocular difference is the difference between the two eyes for a determined ocular parameter. For instance, these interocular differences are commonly induced in monovision technique to correct presbyopia. Monovision is defined as a technique where one eye (the dominant eye) is compensated for distance viewing and the other for near vision^25,26^. Similarly, interocular differences appear in unilateral ocular pathologies, such as cataracts affecting only one eye. After cataract surgery, where the first eye has been operated on and the second eye is awaiting surgery, both visual function and complex visual tasks such as driving are altered^27^. In fact, according to the traffic regulations and visual requirements for driving in most countries, including the UK, France and Spain, individuals are allowed to drive if their binocular visual acuity is greater than or equal to 0.5 or if one eye has an acuity greater than or equal to 0.6 in decimal notation (0.5 for the United States)^28^. On the other hand, an important phenomenon known as intraocular scattering may be responsible for an increase in these interocular differences. When this phenomenon affects one eye more than the other, interocular differences cause a deterioration in binocular visual performance^18,24,29^. An increase in intraocular scattering results in a veiling luminance on the retina which worsens the retinal image quality^24,30^. Intraocular scattering is also implicated in visual disturbances such as disability glare, which reduces contrast sensitivity^31,32^, and night vision disturbances such as halos^33–35^. Furthermore, different levels of intraocular scattering and, consequently, interocular differences, can be simulated by filters such as the Black Pro-Mist 2 fog filter (BPM2) and Bangerter foils^24,36^. The BPM2 filter has been proven to successfully simulate an early cataract^37^ whereas Bangerter foils are widely used to treat amblyopia in children^38,39^.

Taking into consideration the aforementioned publications and arguments, only a few studies have analyzed the effect of interocular differences on a complex visual task such as driving and these have not evaluated the different levels of interocular differences^27,40,41^. Indeed, no study has investigated and reported how a daily visual task such as driving can be affected by interocular differences (which are commonly induced, for example, after cataract surgery on one eye, or when presbyopia is corrected by the monovision technique), and even more so after moderate alcohol consumption. For this reason, it is of interest to study how different degrees of interocular differences (simulated by filters) affect vision and especially driving performance under moderate alcohol consumption conditions equivalent to a BrAC of 0.40 mg/l (or a BAC of 0.08%).

Our hypothesis was that an increase in interocular differences simulated by filters affect complex tasks like driving under the influence of moderate alcohol consumption.

The aim of this study was to assess the influence of moderate alcohol intake (BrAC of 0.40 mg/l) and interocular differences (simulated by a BPM2 filter and a Bangerter foil of 0.8 on the dominant eye), on visual and simulated driving performance.

## Methods

### Subjects

A cohort of 20 participants was included in this crossover study (9 females, 11 males) with a mean age of 26.3 ± 2.9 years and a mean body mass index (BMI) of 22.6 ± 2.9 kg/m^2^; the mean refractive error (spherical equivalent) was − 0.99 ± 1.47 D. The inclusion criteria were as follows: a binocular best-corrected logMAR visual acuity of 0.0 or better; normal stereoacuity at near and distance (40 arcsec or lower); no pathologies or pharmacological treatment that could affect visual performance; no contraindication to alcohol use, a minimum of 24 h without previous alcohol consumption and being a social drinker with a score of less than or equal to 8 on the alcohol use disorders identification test (AUDIT)^42,43^. A complete subjective visual examination was carried out before adding any filter to ensure that the subjects’ refractive errors were correctly compensated. All the participants had to have been in possession of a driving license for at least two years and drive at least 2000 km per year. Finally, sensory ocular dominance was determined by using the line preceding their best visual acuity of the optotype (which corresponds to a less demanding visual acuity) and alternating a + 1.50 D lens in front of each eye under binocular viewing conditions. The sensory-dominant eye was the eye with the positive lens reporting the most blurred vision^44^. Before starting the experiment, all participants had to sign an informed consent form according to the Declaration of Helsinki. The study was approved by the Human Research Ethics Committee of the University of Granada (921/CEIH/2019).

### Filters

Firstly, a Bangerter foil (Ryser Optik, St Gallen, Switzerland) corresponding to grade 0.8 (BF_0.8) was used to simulate degraded retinal image quality. The value of 0.8 corresponds to the theoretical visual acuity in decimal notation obtained using the foil (assuming an initial visual acuity of 1.0 or better); it is well known that Bangerter foils are commonly prescribed to treat amblyopia in children^45,46^. Secondly, the Black Pro-Mist 2 fog filter (BPM2, Tiffen, Hauppauge, NY, USA) was used to simulate an early cataract^36,37^. Both filters (BF_0.8 and BPM2) have been characterized in previous studies using an artificial eye^36^. In practice, each of the two filters was placed on the subject’s sensory-dominant eye. The BPM2 filter was mounted into Knobloch K-2 shooting glasses (Knobloch Optik GmbH. Karlsruhe, Germany) using a filter adapter, which allowed us to fix the lens-holder with the BPM2 filter centered in front of the eye. The BF_0.8 foil was placed over an ophthalmic lens, with no optical power, mounted in an identical optical frame.

### Visual performance

#### Visual acuity and contrast sensitivity

Visual acuity (VA) and contrast sensitivity (CS) were measured using the Pola VistaVision monitor (DMD MedTech, Villarbasse, Turin, Italy). Visual acuity (logMAR notation) and contrast sensitivity (reported as the logCS) were tested binocularly under photopic lighting conditions at a distance of 5.5 m, as well as under mesopic lighting conditions at a distance of 3 m. The luminance background was 60 cd/m^2^. To assess contrast sensitivity, a sequential psychophysical procedure was used. For each spatial frequency, eight different contrast levels were proposed. The participants were asked to indicate the orientation of the sinusoidal grid (right, left or vertical) for different decreasing contrasts (from highest to lowest contrast). Eight different spatial frequencies were evaluated: 0.75, 1.5, 3, 6, 12, and 18 cycles per degree (cpd). Subsequently, under each condition, we converted the contrast sensitivity for these spatial frequencies in logarithmic scale (logCS) The higher the contrast sensitivity value (in logarithmic scale), the better the binocular visual performance.

#### Stereoacuity

Stereopsis is the most advanced degree of binocular vision and allows depth perception. Stereopsis was evaluated at a distance of 5.5 m using the differentiated stereo D8 polarized test of the Pola VistaVision monitor (DMD MedTech, Villarbasse, Turin, Italy) by means of polarized vertical lines displayed on the monitor^47^. Stereopsis was quantified according to distance stereoacuity under photopic conditions, in such a way that the higher the stereoacuity value, the worse the stereopsis. For each disparity test, 5 vertical lines were displayed on the monitor, one of which presented disparity. The task of the subjects, who wore polarizing glasses, was to recognize which of the 5 lines was perceived stereoscopically. A total of 8 disparities (from 300 to 10 arcseconds) were evaluated.

#### Visual discrimination capacity

**Figure 1:**
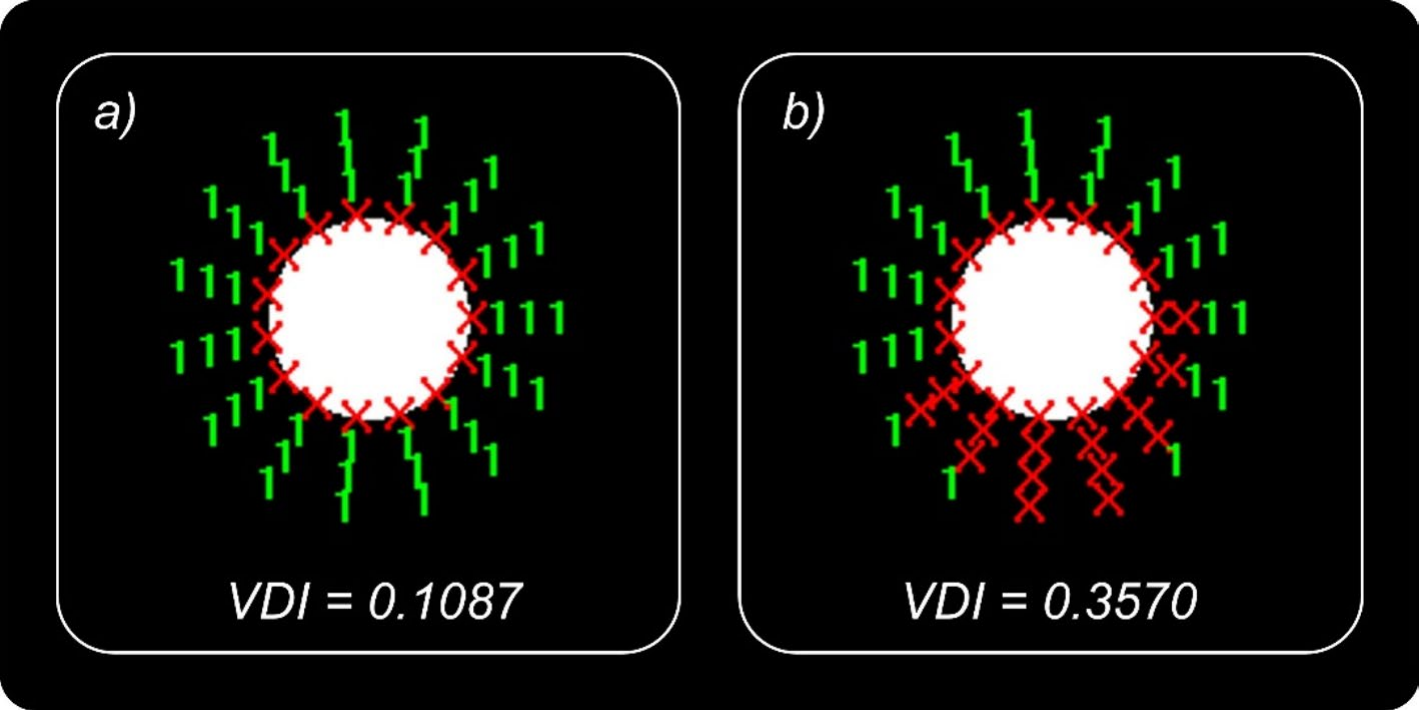
Two examples of graphical results of the Halo test: (**a**) low halo influence (low VDI value); (**b**) strong halo (higher VDI value). Red X in the graph: undetected peripheral stimulus; green number 1: peripheral stimulus detected.

We evaluated visual discrimination capacity using the Halo test, which is based on the Halo v1.0 freeware (University of Granada, Granada, Spain; http://hdl.handle.net/10481/5478). The test consists of detecting peripheral stimuli (1-pixel radius subtending 0.02 degrees from the observer’s position) presented randomly around a central stimulus (30-pixel radius subtending 0.46 degrees from the observer’s position) under low light conditions. The test was performed at a distance of 2.5 m. Each peripheral stimulus was presented in one of the four possible positions per semi-axis, out of a total of 15 semi-axes (Fig. 1). The higher-luminance central stimulus is responsible for the perception of halos and other night vision disturbances such as glare or starbursts. During the test, the participants had to detect the peripheral light stimuli. At the end of the test, a visual disturbance index (VDI) was obtained to quantify the participant’s positive dysphotopsia (phenomena of light, such as bright lines, glare or halos). This index is calculated by taking into account undetected stimuli versus total stimuli presented to the subject: the higher the VDI the worse the visual discrimination capacity of the participant and, therefore, the greater the halo perceived. This parameter has been widely employed in clinical applications, such as ocular pathologies^21,48,49^, refractive surgery^50–52^, and monovision technique^53^ to quantify dysphotopsias and night-vision disturbances.

### Assessment of ocular parameters

#### Retinal-image quality

The OQAS II (Optical Quality Analysis System II, Visometrics, Terrassa, Spain) double-pass device was used to assess retinal image quality. This device has been widely used and validated in clinical practice^48,54,55^. We used the main objective parameter to evaluate retinal-image quality, namely the objective scatter index (OSI). The OSI quantifies the intraocular scattering in the outer part of the double-pass image for an artificial pupil size of 4 mm. It takes into account the light intensity within an annular area of between 12 and 20 arc min (near-angle scattering) relative to the central peak of the double-pass image in such a way that the higher the OSI value, the greater the intraocular scattering affecting retinal image quality. A normal value for this parameter would be less than 1.0^56^.

#### Straylight

The intraocular straylight was measured using a C-Quant device (Oculus GmbH, Wetzlar, Germany), which employs a compensation comparison method; this device is used extensively in clinical practice^37,57,58^. In this visual test, the participants had to recognize which of two central semicircular fields flickered the most, in several iterations. One of the two flickers depends on straylight and the other on the combination of straylight and compensation light^59^. At the end of the test, the ocular parameter log(s) (logarithm of the straylight) is obtained. This parameter is defined by the ratio between the scattered and non-scattered light so that the higher this value is, the greater the intraocular straylight and, therefore, the greater the visual quality deterioration. This parameter depends on age; the normal value for young healthy eyes is about 0.90^60^. In each condition and monocularly (for both eyes), only measurements with a standard deviation of less than 0.08 were taken into account in the data analysis.

### Interocular differences

Interocular differences (ID) are defined by the variation between the dominant and non-dominant eye for given ocular parameters. In this study, we calculated the ID (in absolute values) for the OSI and log(s)^24,29,33^; the ID was determined under natural conditions, and successively, between the dominant eye wearing the corresponding filter (BPM2 or BF_0.8) and the non-dominant eye under natural conditions (no filter). Considering these filter conditions, the IDs were calculated with (BrAC of 0.40 mg/l) and without alcohol consumption (baseline).

### Driving performance

**Figure 2:**
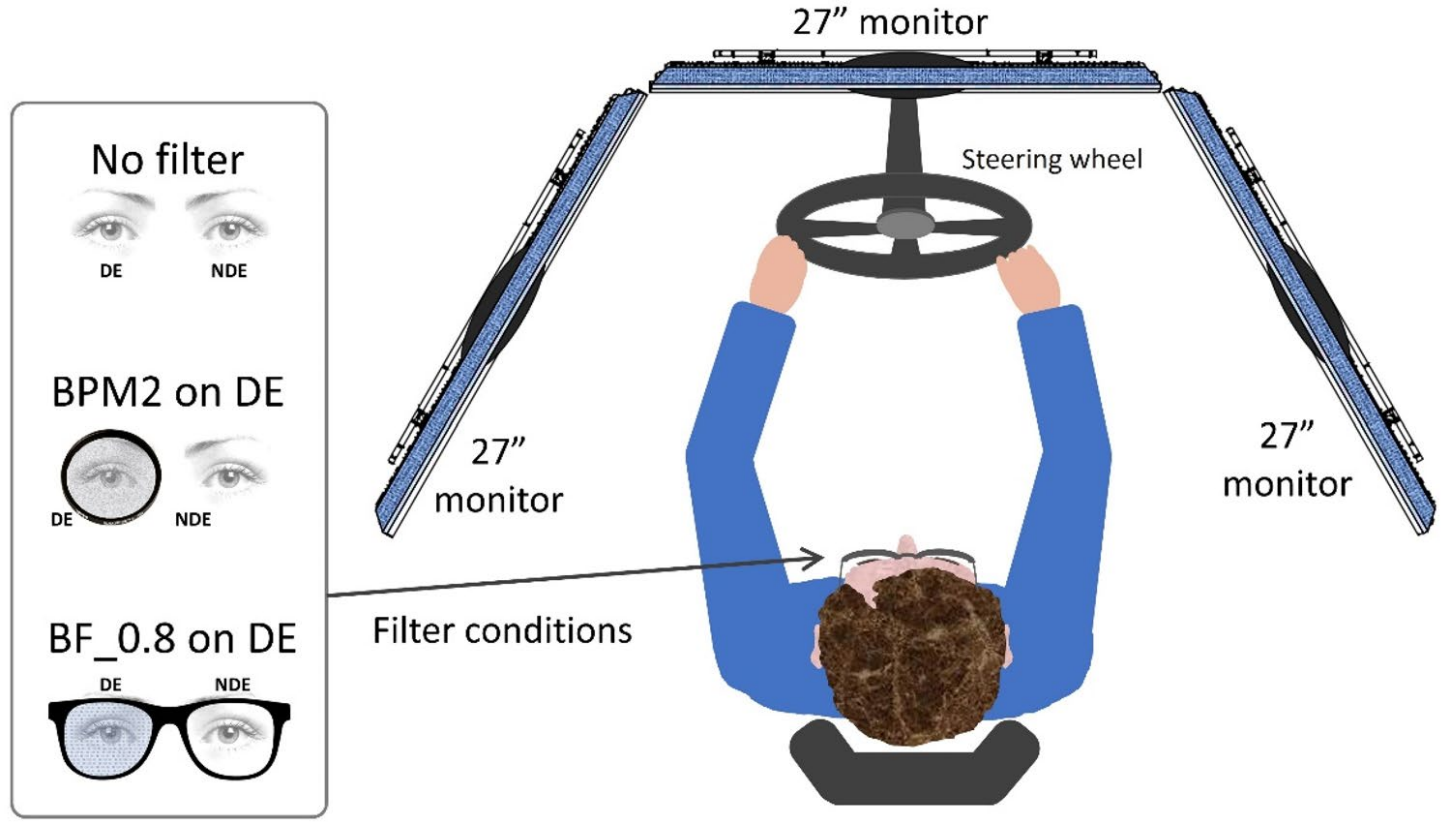
Setup of the driving simulator with the different binocular visual conditions. BPM2 on DE: Black Pro-Mist 2 fog filter on the dominant eye; BF_0.8 on DE: Bangerter foil of 0.8 on the dominant eye; NDE: non-dominant eye.

To assess simulated driving performance, we used the Simax Driving Simulator v4.0.8 Beta software (SimaxVirt S.L., Pamplona, Spain). This comprises three high definition 27″ screens (resolution of 1920 × 1080 pixels) with a 180° field of view, a car seat, a Logitech G27 Racing Wheel (Logitech International SA, Lausanne, Switzerland) including a steering wheel, gearshift (six speeds and reverse), and three pedals (accelerator, brake, and clutch) (Fig. 2). The driving simulator has proved to be a valid tool for analyzing driving-related parameters^61,62^. In this study, simulated driving was performed under photopic lighting conditions, representing the best conditions for driving performance^63,64^; the driving scenario was also simulated in daylight and good weather conditions.

The complete route was approximately 12.5 km long and included three main sections simulating different common road environments with moderate traffic. The first section was a 4.5 km long dual carriageway with a speed limit of 120 km/h (current maximum speed limit in Spain). The second section was a 6 km single carriageway mountain road with a speed limit ranging from 40 to 90 km/h. The third section was a 2 km inner-city circuit with a speed limit of 40–50 km/h.

The participants came to the laboratory four times in four separate weeks. During the simulated driving tests, all the participants were instructed to drive normally and to respect traffic laws. In the first two visits (week 1 and 2), the participants completed two training sessions, with each session consisting of one full lap of the driving scenario (12.5 km), to acclimatize them and familiarize them with the driving simulator^65^. During the baseline session (week 3) and the after-alcohol consumption (aAC) session (week 4), the participants completed one full lap of the driving scenario and performed all the visual tests. Only the latter two sessions were considered for the analysis of the results. The participants performed the visual tests and the simulated driving scenario in a random order.

The following variables were measured to assess driving performance: mean speed (MS, km/h), distance traveled invading the shoulder (DTIS, m), distance traveled invading the opposite lane (DTIOL, m), total distance traveled outside the lane (TDTOL, m), standard deviation of the lateral position (SDLP, m), reaction time (RT, s), and number of collisions (times). The reaction time was calculated as the interval between the instant the brake lights turned on in the preceding car and the moment the driver pressed the brake pedal. The SDLP is a variable that measures steering stability in such a way that the higher the SDLP, the lower the stability of the car in the center of the lane. The SDLP is a valid indicator of driving under the influence of alcohol^7,8,66,67^.

### Experimental procedure

In addition to the two initial training sessions with the driving simulator, the participants took part in two sessions that included all the tests: a first session with no alcohol consumption (baseline) and a second session after alcohol consumption (aAC), both under all filter conditions. During each session, visual (VA, CSF, VDI, and distance stereoacuity) and simulated driving performance were measured binocularly. Ocular parameters (OSI and log(s)) were measured monocularly and then the interocular differences were calculated for the two parameters. All the visual tests and driving sessions were randomized in the two experimental sessions (baseline and aAC) to avoid learning effects. Finally, the two sessions were performed one week apart to limit any order effect.

In the alcohol consumption session, the participants consumed a mixed alcoholic beverage (67% orange juice and 33% vodka). The breath alcohol content (BrAC), defined in milligrams of ethanol per liter of exhaled air (mg/l), was measured using the Alcotest 6810 breath analyzer (Dräger Safety AG& Co. Lubeck, Germany) which provides good reproducibility^68^. The required BrAC was 0.40 mg/l corresponding to a moderate alcohol content and the legal limit for driving in many countries^1^. To calculate the quantity of alcohol ingested by each participant, an improved version of the Widmark formula was used^69^. Each participant consumed the corresponding dose of alcohol within a time period of 30–40 min. During the experimental session after alcohol consumption, the BrAC was measured every 20 min to check the stability of the BrAC at 0.40 mg/l. The mean BrAC obtained in this research for all participants was 0.40 ± 0.04 mg/l. They were informed about the alcohol consumption to test them in a common real-world situation.

### Statistical analysis

For the data analysis, we used the SPSS 26.0 software package (SPSS Inc., Chicago, IL). We analyzed the normal distribution of all parameters (Shapiro–Wilk test). In the case of normal distribution, a t-test for two-sided alternatives (t) was performed to compare each visual and driving variable used (with the same filter condition) between the two experimental conditions (baseline and aAC). Similarly, the Wilcoxon signed rank test (Z) was used in the case of non-normal distribution. Comparing the different filter and experimental conditions (baseline and after alcohol consumption), an ANOVA test for repeated measures with Bonferroni correction was used to analyze the interactions under the experimental and filter conditions of the visual and driving parameters. Otherwise, for interocular differences, a Friedman test using a two-way ANOVA was run with Bonferroni correction. Finally, Spearman's rank correlation coefficient (or Spearman’s ρ) was calculated between the overall interocular differences score (OIDS) of the ocular parameters (OSI and log(s)), the overall visual performance score (OVPS) including all measured binocular visual function parameters (VA, logCS for all the spatial frequencies measured, VDI, and distance stereoacuity), and the overall driving performance score (ODPS) including all driving variables (MS, SDLP, DTIS, DTIOL, TDTOL, RT and collisions). The three overall scores (OIDS, OVPS, and ODPS) were obtained by averaging the z-scores of all the variables included in each overall score and the different experimental conditions measured such that: the higher the OIDS, the higher the interocular differences; the higher the OVPS, the higher the visual performance; and finally, the higher the ODPS, the worse the driving performance. The Z-score measures how many standard deviations an individual value lies away from the group mean and has been used in a number of studies^7,8,24,49^. A statistical significance level of 95% was applied for all tests (p < 0.05).

## Results

We studied the mean values of contrast sensitivity (in logarithmic scale) at different spatial frequencies (0.75, 1.5, 3, 6, 12 and 18 cpd) under various filter and experimental conditions (baseline and aAC) as shown in Table 1.

**Table 1:** Mean values and standard deviations for all spatial frequencies of the logCS (logarithm of the contrast sensitivity) under the different experimental and filter conditions.

LogCS	Filter	Baseline	aAC
0.75 cpd	No filter	2.11 ± 0.00	2.08 ± 0.06
	BPM2 on DE	2.11 ± 0.00	2.08 ± 0.07
	BF_0.8 on DE	2.11 ± 0.03	2.07 ± 0.15
1.5 cpd	No filter	2.23 ± 0.00	2.22 ± 0.03
	BPM2 on DE	2.22 ± 0.03	2.22 ± 0.05
	BF_0.8 on DE	2.23 ± 0.00	2.20 ± 0.06
3 cpd	No filter	2.34 ± 0.00	2.33 ± 0.03
	BPM2 on DE	2.34 ± 0.03	2.29 ± 0.10
	BF_0.8 on DE	2.34 ± 0.00	2.31 ± 0.05
6 cpd	No filter	2.38 ± 0.09	2.34 ± 0.15
	BPM2 on DE	2.35 ± 0.12	2.22 ± 0.16
	BF_0.8 on DE	2.31 ± 0.13	2.23 ± 0.19
12 cpd	No filter	2.18 ± 0.08	2.09 ± 0.13
	BPM2 on DE	2.03 ± 0.23	1.91 ± 0.25
	BF_0.8 on DE	2.07 ± 0.18	1.95 ± 0.26
18 cpd	No filter	1.68 ± 0.20	1.37 ± 0.26
	BPM2 on DE	1.31 ± 0.26	1.06 ± 0.32
	BF_0.8 on DE	1.31 ± 0.35	1.18 ± 0.34

For the 3 cpd spatial frequency, there was a significant negative effect with alcohol consumption across all the filter conditions (F(1,19) = 9.206, p = 0.007, η_p_^2^ = 0.326). For the 6 cpd spatial frequency, significant effects were observed under alcohol consumption (F(1,19) = 11.969, p = 0.003, η_p_^2^ = 0.386) and filter conditions (F(1,19) = 6.404, p = 0.004, η_p_^2^ = 0.252). In this specific spatial frequency, a significant interaction was found between alcohol consumption and filter conditions, suggesting that filter conditions had an even greater impact under the influence of alcohol (F(1,19) = 3.741, p = 0.044, η_p_^2^ = 0.165).

The highest spatial frequencies (12 and 18 cpd) showed significant deterioration under alcohol consumption (for 12 cpd, F(1,19) = 9.019, p = 0.007, η_p_^2^ = 0.322; for 18 cpd, F(1,19) = 36.500, p < 0.001, η_p_^2^ = 0.658) and filter conditions (for 12 cpd, F(1,19) = 14.967, p < 0.001, η_p_^2^ = 0.441; for 18 cpd, F(1,19) = 32.089, p < 0.001, η_p_^2^ = 0.628).

It can therefore be seen that moderate alcohol consumption (from 3 to 18 cpd) and filter conditions (from 6 to 18 cpd) negatively affected contrast sensitivity.

Regarding driving performance, Table 2 presents the mean values for all simulated driving parameters measured under the different experimental and filter conditions.

**Table 2:** Mean values and standard deviations for all simulated driving parameters measured at the baseline (Bas) and after alcohol consumption (aAC; BrAC = 0.40 mg/l), as well as for different filter conditions.

		No filter	BPM2 on DE	BF_0.8 on DE
Dual carriageway	MS (km/h)	Bas: 116.3 ± 4.9 aAC: 126.7 ± 9.2	Bas: 116.6 ± 4.8 aAC: 124.4 ± 13.5	Bas: 117.2 ± 5.8 aAC: 127.2 ± 16.0
	SDLP (m)	Bas: 0.508 ± 0.105 aAC: 0.707 ± 0.185	Bas: 0.539 ± 0.128 aAC: 0.704 ± 0.205	Bas: 0.532 ± 0.099 aAC: 0.757 ± 0.237
	DTIS (m)	Bas: 69.2 ± 85.2 aAC: 238.1 ± 238.4	Bas: 84.5 ± 74.9 aAC: 315.0 ± 375.4	Bas: 96.2 ± 123.2 aAC: 267.7 ± 178.8
Two-lane mountain road	MS (km/h)	Bas: 56.1 ± 1.2 aAC: 58.0 ± 4.9	Bas: 56.3 ± 1.7 aAC: 56.7 ± 5.6	Bas: 56.2 ± 2.8 aAC: 59.3 ± 5.2
	SDLP (m)	Bas: 0.551 ± 0.075 aAC: 0.764 ± 0.159	Bas: 0.571 ± 0.101 aAC: 0.775 ± 0.217	Bas: 0.574 ± 0.092 aAC: 0.830 ± 0.227
	DTIS (m)	Bas: 51.5 ± 61.2 aAC: 251.5 ± 292.0	Bas: 95.7 ± 95.8 aAC: 313.2 ± 441.7	Bas: 106.3 ± 170.5 aAC: 289.5 ± 236.8
	DTIOL (m)	Bas: 326.3 ± 285.3 aAC: 562.6 ± 442.5	Bas: 326.3 ± 290.5 aAC: 587.9 ± 518.8	Bas: 354.6 ± 405.1 aAC: 710.1 ± 601.0
	TDTOL (m)	Bas: 377.7 ± 266.3 aAC: 814.1 ± 486.6	Bas: 422.1 ± 272.6 aAC: 901.0 ± 659.0	Bas: 460.9 ± 382.1 aAC: 999.6 ± 686.3
	RT (s)	Bas: 0.83 ± 0.13 aAC: 0.94 ± 0.15	Bas: 0.92 ± 0.11 aAC: 1.01 ± 0.13	Bas: 0.94 ± 0.16 aAC: 1.02 ± 0.12
Inner city	MS (km/h)	Bas: 30.9 ± 4.8 aAC: 35.9 ± 4.3	Bas: 32.6 ± 6.0 aAC: 36.4 ± 7.5	Bas: 32.1 ± 6.1 aAC: 35.2 ± 6.8
	SDLP	Bas: 0.892 ± 0.218 aAC: 1.007 ± 0.234	Bas: 0.936 ± 0.200 aAC: 1.213 ± 0.316	Bas: 0.984 ± 0.270 aAC: 1.162 ± 0.296
Event	Collisions (times)	Bas: 0.0 ± 0.0 aAC: 4.3 ± 3.9	Bas: 1.1 ± 1.3 aAC: 6.7 ± 7.4	Bas: 0.9 ± 1.1 aAC: 5.5 ± 3.5

MS, mean speed; SDLP, standard deviation of the lateral position; DTIS, distance traveled invading the shoulder; DTIOL, distance traveled invading the opposite lane; TDTOL, total distance traveled outside the lane; RT, reaction time and number of collisions.

For the dual carriageway section, all driving parameters were significantly impaired after alcohol consumption under all filter conditions (for mean speed, F(1,19) = 16.016, p = 0.001, η_p_^2^ = 0.457; for SDLP, F(1,19) = 31.786, p < 0.001, η_p_^2^ = 0.626 and for DTIS, F(1,19) = 25.368, p < 0.001, η_p_^2^ = 0.572).

For the two-lane mountain road section, all the driving parameters deteriorated after alcohol consumption in all filter conditions (for SDLP, F(1,19) = 47.321, p < 0.001, η_p_^2^ = 0.714; for DTIS, F(1,19) = 11.077, p = 0.004, η_p_^2^ = 0.368; for DTIOL, F(1,19) = 27.210, p < 0.001, η_p_^2^ = 0.589; for TDTOL, F(1,19) = 27.719, p < 0.001, η_p_^2^ = 0.593 and for RT, F(1,19) = 20.065, p < 0.001, η_p_^2^ = 0.514), except for mean speed (F(1,19) = 4.363, p = 0.050, η_p_^2^ = 0.187). In addition, under the filter conditions, we observed a statistical deterioration compared to the no-filter condition in terms of reaction time (F(1,19) = 8.898, p < 0.001, η_p_^2^ = 0.319).

For the inner-city section, all the driving parameters were statistically impaired after alcohol consumption (for mean speed, F(1,19) = 14.002, p = 0.001, η_p_^2^ = 0.424 and for SDLP, F(1,19) = 12.569, p = 0.002, η_p_^2^ = 0.398) under all filter conditions. Similarly, under the filter conditions, we found a statistical deterioration compared to the no-filter condition in terms of SDLP (F(1,19) = 3.500, p = 0.040, η_p_^2^ = 0.156).

Over the three sections, after alcohol consumption the number of collisions increased in all filter conditions (F(1,19) = 25.962, p < 0.001, η_p_^2^ = 0.577). Similarly, under filter conditions, all filters placed on the dominant eye resulted in an impairment compared to no filter (F(1,19) = 4.862, p = 0.013, η_p_^2^ = 0.204).

In summary, alcohol consumption and filter conditions negatively affected driving performance (by means of reaction time, SDLP and number of collisions). However, no interaction effect between alcohol consumption and filter condition was found in driving performance, indicating that adding the filter does not further deteriorate subjects when they are under the influence of alcohol F(1,19) = 1.296, p = 0.285, η_p_^2^ = 0.064).

Table 3 shows the interocular differences for OSI and log(s) for all the filters analyzed and under the different experimental conditions. For all filter conditions, comparing the baseline and aAC, no change was found for interocular differences in log(s) and OSI. Nevertheless, by comparing the no-filter with other filter conditions, significant increases were observed (for baseline, log(s): χ^2^(2) = − 0.975, p = 0.006, and OSI: χ^2^(2) =  − 0.675, p = 0.028; for aAC, log(s): χ^2^(2) =  − 1.150, p = 0.001, and OSI: χ^2^(2) =  − 0.700, p = 0.027). For OSI, in both experimental conditions, statistically significant increases were also found between BPM2 on DE and BF_0.8 on DE (baseline: χ^2^(2) =  − 1.325, p < 0.001; and aAC: χ^2^(2) =  − 1.150, p = 0.001) contrary to the results for log (s) (p > 0.05).

**Table 3:** Mean values of the interocular differences for the ocular parameters analyzed.

Interocular differences	Filter	Baseline	aAC	t/Z; p-value
OSI	No filter	0.19 ± 0.20	0.20 ± 0.14	Z(19) = − 0.854; p = 0.393
	BPM2 on DE	0.36 ± 0.23	0.56 ± 0.42	Z(19) = − 1.771; p = 0.076
	BF_0.8 on DE	3.47 ± 0.95	3.21 ± 1.18	Z(19) = − 0.990; p = 0.322
log(s)	No filter	0.07 ± 0.09	0.09 ± 0.08	Z(19) = − 1.124; p = 0.261
	BPM2 on DE	0.24 ± 0.11	0.28 ± 0.16	Z(19) = − 1.140; p = 0.254
	BF_0.8 on DE	0.30 ± 0.20	0.30 ± 0.13	Z(19) = − 0.224; p = 0.823

Objective scatter index (OSI) and straylight (log(s)) under the different experimental and filter conditions. Standard deviations and statistical results (t/Z) with the p-values are included.

Thus, in both experimental conditions, the visual deterioration filters increased the interocular differences compared to the no-filter condition.

**Figure 3:**
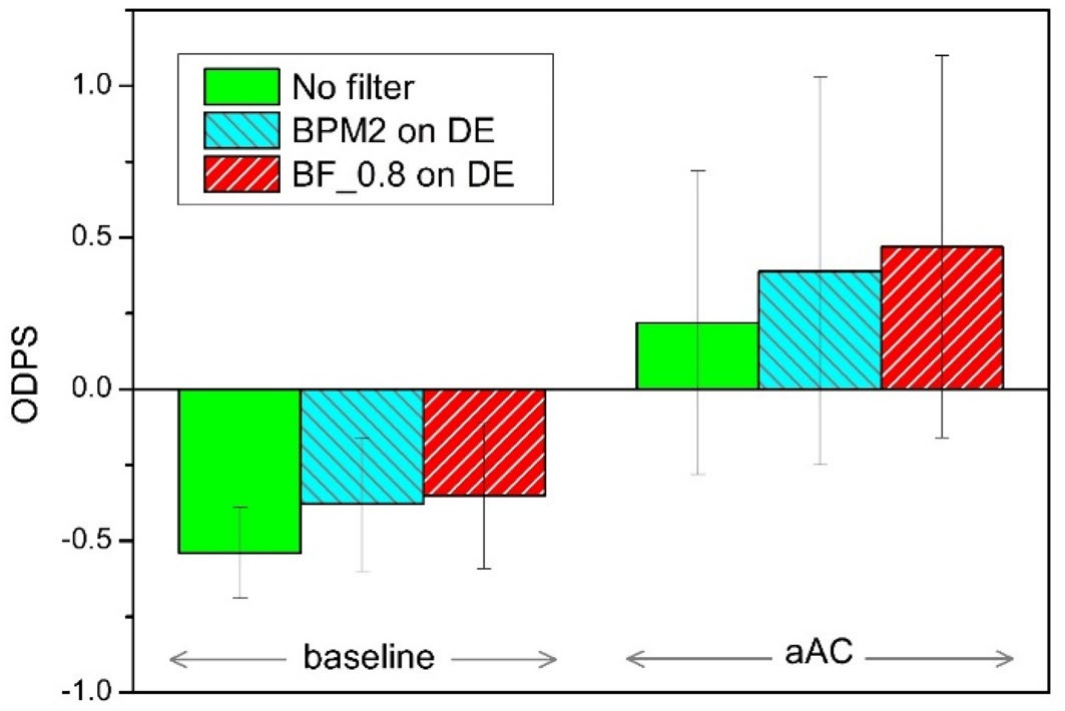
The overall driving performance score (ODPS) under the two experimental and filter conditions.

Figure 3 shows the overall driving performance score (ODPS) under the different experimental and filter conditions in bar graph form. Comparing the baseline and aAC, the ODPS was impaired under all filter conditions after alcohol consumption (t(19) = − 5.262, p < 0.001). Under the baseline condition, ODPS was significantly impaired by all the filters compared to the no-filter condition (F(2,19) = 11.232, p = 0.003). Similarly, after alcohol consumption, the ODPS was significantly deteriorated in BF_0.8 on DE conditions compared to no filter (p = 0.048).

Therefore, considering all the driving parameters (mean speed, SDLP, DTIS, DTIOL, TDTOL, reaction time, and collisions), it can be seen that both alcohol consumption and penalizing filters negatively affected driving performance.

**Figure 4:**
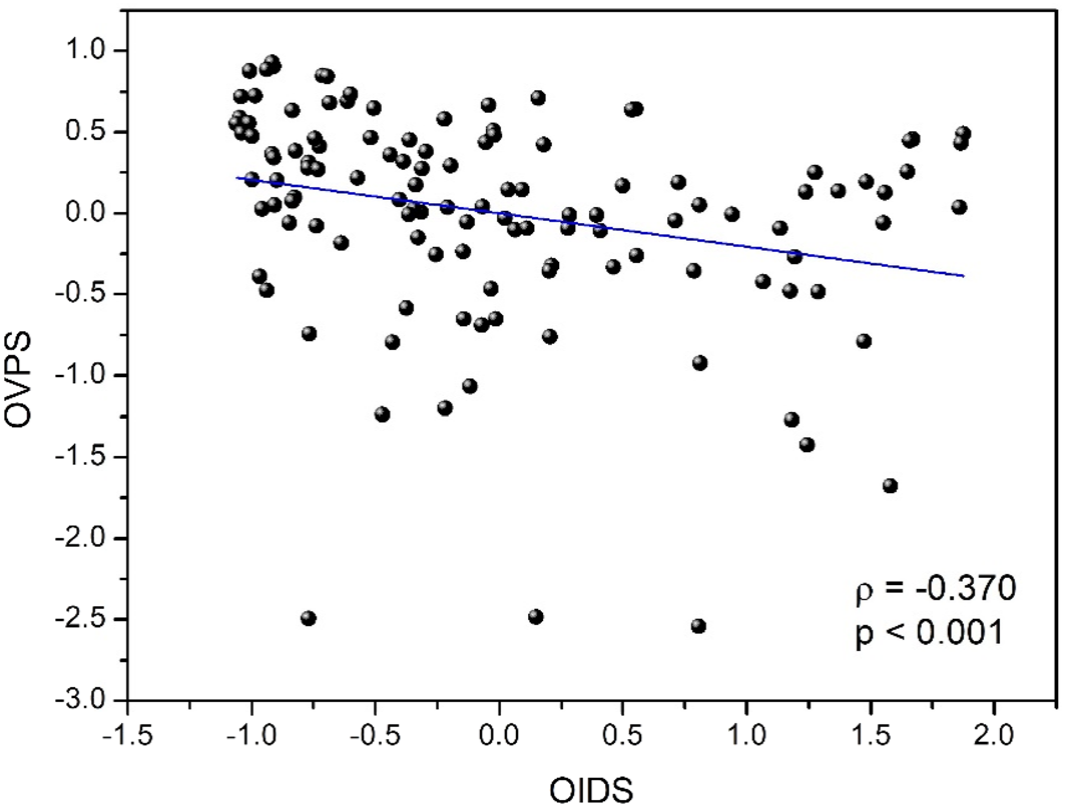
The overall visual performance score (OVPS) as a function of the overall interocular differences score (OIDS) under the two experimental and filter conditions.

Figure 4 presents the relationship between the overall visual performance score (OVPS) for visual function and the overall interocular difference score (OIDS) for ocular parameters. A significant negative correlation was found between these two overall scores (ρ = − 0.370, p < 0.001) considering all filters and under the two experimental conditions (baseline and aAC). Thus, the greater the interocular differences in ocular parameters, the poorer the visual performance. As a result, the improvement in binocular visual performance was related to a decrease in interocular differences.

**Figure 5:**
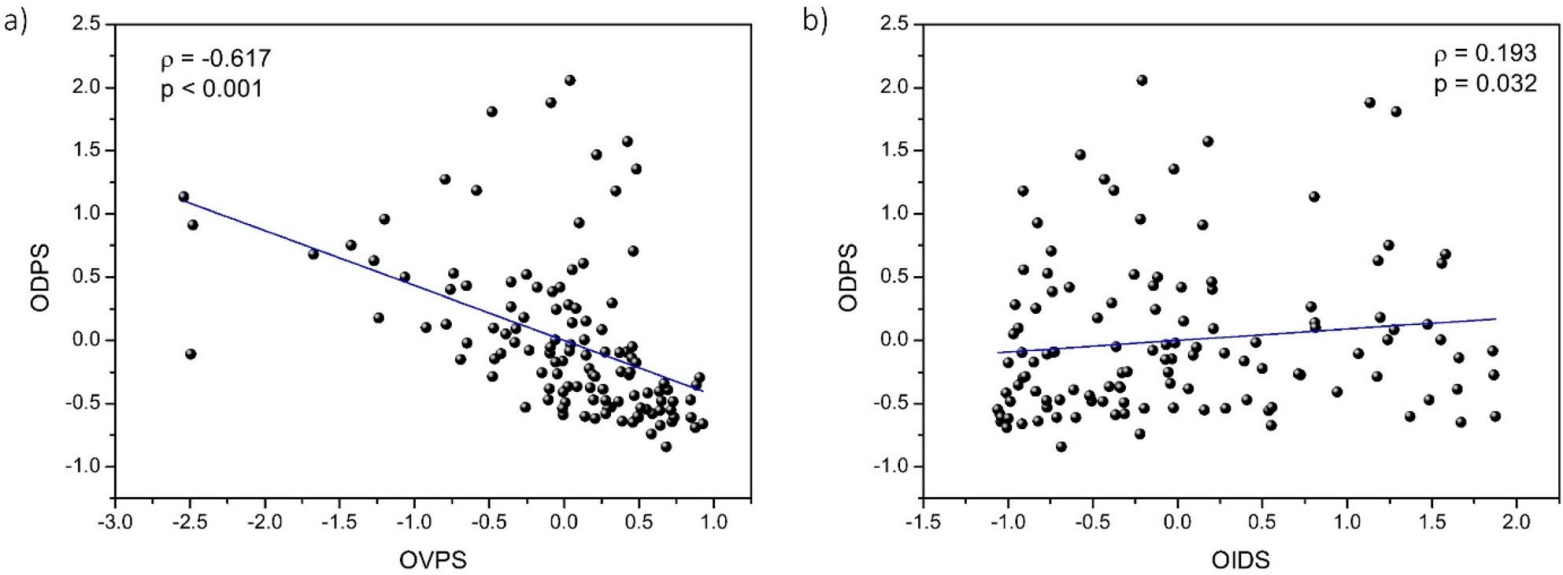
The overall driving performance score (ODPS) as a function of (**a**) the overall visual performance score (OVPS), and (**b**) the overall interocular differences score (OIDS), under the two experimental and filter conditions.

The relationship between the ODPS (including all the driving parameters measured) and OVPS (all the visual function parameters comprised) under the different filter and experimental conditions is shown in Fig. 5a. A significant negative correlation was found between the two overall scores (ρ = − 0.617, p < 0.001), in such a way that the higher the ODPS (worse driving performance), the lower the OVPS (worse visual performance). As a result, increased deterioration in binocular visual performance was associated with poorer driving performance.

In addition, the relationship between the ODPS for driving parameters and OIDS for ocular parameters (log(s) and OSI) under the filter and experimental conditions is depicted in Fig. 5b. Despite the data variability, a significant positive correlation was obtained between these two overall scores (ρ = 0.193, p = 0.032), in such a way that the greater the interocular differences, the worse the driving performance. As a result, better driving performance is related to decreased interocular differences in ocular parameters.

## Discussion

This study provides a comprehensive perspective of the negative impacts of moderate alcohol consumption (BrAC of 0.40 mg/l) and interocular differences (induced by filters BPM2 and BF_0.8 on the dominant eye) on binocular visual performance (including VA, logCS for all the spatial frequencies measured, VDI, and distance stereoacuity), as well as on driving performance (mean speed, SDLP, distance traveled outside the lane, reaction time, and collisions). In addition, this is the first experimental study to analyze the effects of interocular differences (induced by filters simulating different levels of intraocular scattering) on driving performance under the influence of moderate alcohol consumption.

Firstly, contrast sensitivity measurements confirmed a significant deterioration (from 3 to 18 cpd) in binocular visual function with a moderate alcohol intake (BrAC of 0.40 mg/l). Watten and Lie^16^ also found a significant deterioration for the highest spatial frequencies (12 and 18 cpd) in contrast sensitivity for a mean BrAC of 0.25 and 0.50 mg/l, as did Pearson and Timney^70^ also for a BrAC of 0.30 mg/l. As reported by Casares-Lopez et al.^17^, for a mean BrAC of 0.33 mg/l, mean binocular contrast sensitivity was impaired with moderate alcohol consumption (6 and 12 cpd). In the present study, we have confirmed significant deteriorations in contrast sensitivity under the influence of a moderate alcohol consumption (from 3 to 18 cpd) and filter conditions (from 6 to 18 cpd). In addition, our findings determined an important interaction effect between alcohol and the filter suggesting that the addition of the filter exacerbate the subjects' condition when they are exposed to alcohol for the 6 cpd spatial frequency. Contrast sensitivity is an important visual function that could be considered a regular visual requirement for a driving license^7,27,71,72^.

Secondly, in terms of driving performance, we found that moderate alcohol consumption significantly impairs driving skills, especially mean speed, SDLP, distance traveled outside the lane, reaction time, and collisions. Consistent with other studies^7,8,14^, we observed faster driving under the influence of moderate alcohol intake. Driving parameters such as SDLP^13,73^ and distance traveled outside the lane^7,8^ increased under the influence of alcohol (equivalent to a BrAC of 0.40 mg/l), worsening driving performance. This highlights car-control difficulties and, therefore, road safety issues. Furthermore, in our study, reaction time (the capacity of the psychomotor reflex to respond in time to a simulated driving situation) increased, showing slower reflexes when reacting to a situation of driving risk. Specifically, when evaluating driving performance, the number of collisions rose after alcohol consumption indicating lower levels of road safety. This is in line with other studies assessing BrAC between 0.25 and 0.40 mg/l^8,10,11,74^, which noted that alcohol can induce somnolence and slower attention^10,11,75^. Khan and Timney^2^ found that this decline in driving performance is due to decreased neural processing and reduced temporal visual processing resulting in a failure to process information correctly. Unlike the aforementioned studies, we induced a visual deterioration in the dominant eye using filters (BPM2 and Bangerter foil of 0.8) to simulate an ocular pathology, such as a cataract. In general, under the experimental conditions (baseline and aAC), significant impairments in driving performance were observed with BPM2 (simulating an early cataract) and BF 0.8 on the dominant eye, compared to the no-filter condition. Molina et al.^40^ found a significant deterioration in driving performance when one eye was penalized with a 0.2 Bangerter foil, suggesting that drivers adopt self-regulatory strategies when their binocular vision is compromised. Using the same driving simulator, Ortiz-Peregrina et al.^49^ highlighted the fact that driving performance is also significantly worse in drivers with bilateral cataracts, as reflected by a notable deterioration in lane keeping. Additionally, Meuleners et al.^27^ observed that the rate of crashes decreased significantly by 36% after the first eye surgery and 47% after the second operation compared to the rate prior to cataract surgery on the first eye. Their findings suggest the importance of timely first and second eye cataract surgery to ensure driver safety, especially as older drivers wait for second eye cataract surgery. In contrast to the aforementioned studies, we simulated an early cataract (BPM2) on the dominant eye and found significantly altered driving performance compared to the no-filter condition both before and after alcohol consumption, highlighting an increase in the number of collisions. An important contribution of this work is that it is the first study to assess driving performance for added of two important conditions related to driving safety, i.e., alcohol consumption and visual deterioration (simulating an early cataract or a stronger forward scattering effect). We have observed a statistically significant influence of alcohol consumption as well as the presence of the filter. Furthermore, the absence of an interaction effect between alcohol and the filter suggests that the addition of the filter does not exacerbate the subjects' condition when they are exposed to alcohol.

Thirdly, interocular differences between the two ocular parameters (OSI and log(s)) increased significantly and gradually, from the baseline to the BF_0.8 on the dominant eye. Interocular differences are commonly generated in emmetropization techniques for presbyopia, such as monovision. Evans et al.^76^ noted that the main issues encountered in this technique are related to the suppression of the blurred image when driving at night and binocular vision degradation, for example, in stereopsis. Similarly, Castro et al.^53^ showed a deterioration in binocular visual performance including a greater perception of halos and lower contrast sensitivity when inducing anisocoria and with any add power after simulating monovision conditions. In this study, we investigated the effect of moderate alcohol consumption on interocular differences. We found that visual impairment due to alcohol consumption could be expected to affect both eyes equally, the interocular differences being maintained before and after alcohol consumption. In a previous study^18^, we showed similar results taking into account the same ocular parameters and alcohol consumption condition where interocular differences significantly increased due to visual deterioration filters (BPM2 and BF_0.8 on the dominant eye). The two ocular parameters were considered due to their importance for quantifying intraocular scattering and straylight. In addition, these parameters are suitable for evaluating different degrees of simulated cataract using specific filters. De Wit et al.^37^ proved that the BPM2 fog filter approximates an early-stage cataract in terms of straylight quantified by the log(s) parameter, using the same device as in this study. To objectively quantify intraocular scattering (OSI), Artal et al.^56^ classified this ocular parameter and compared it to a LOCSIII chart classification to categorize the different degrees of cataract. Subsequently, in relation to the previous OSI classification, Castro et al.^36^ determined that the BPM2 filter correctly simulates the OSI of an early cataract while the BF_0.8 filter mimics that of a mild cataract.

Finally, in this study, we established several significant correlations that improve the understanding of the mutual influence between visual and driving performance and interocular differences under different conditions (filters and moderate alcohol consumption). We confirmed a correlation between visual performance in visual function and interocular differences in ocular parameters (ρ = − 0.370, p < 0.001), such that the greater the interocular differences in the ocular parameters studied, the poorer the visual performance. In a recent study^18^, we also found a correlation (ρ = − 0.390) between the overall binocular summation of several visual functions (VA, CS, and VDI) and interocular differences (including intraocular scattering, retinal-image quality, and straylight) when considering the same filter conditions and alcohol consumption. In contrast to that study, we have included stereopsis, which, as is well established, is an essential aspect of binocular vision and, therefore, visual performance. In addition, a high correlation coefficient (ρ = − 0.617, p < 0.001) was obtained by comparing the overall driving performance (including mean speed, SDLP, distance traveled invading the shoulder, distance traveled invading the opposite lane, total distance traveled outside the lane, reaction time in all the experimental and filter conditions) as a function of the overall visual performance (including VA in logMAR, logCS for the different spatial frequencies studied, VDI and distance stereopsis in all the experimental and filter conditions). Thus, deteriorated driving performance and unsafe driving are strongly associated with deteriorated visual performance under all the experimental conditions analyzed (filters and alcohol). In one of our previous studies^8^, we also found a correlation (ρ = 0.390, p < 0.001) between visual and driving deterioration after moderate alcohol consumption (BrAC of 0.40 mg/l). The greater the visual deterioration (including all the variables of the vergence system, visual acuity, and near and far stereoacuity), the greater the driving deterioration (including similar driving variables to those in this study). However, no simulated visual impairment was induced, unlike the present study. Casares et al.^7^ also highlighted that lower binocular contrast sensitivity is inherent in worsening driving under alcohol consumption conditions. As far as we know, this is the first study to prove a comprehensive relationship between these two parameters by inducing visual degradation (filters) in combination with alcohol consumption. We determined another important and novel correlation between driving performance and interocular differences (ρ = 0.193, p = 0.032): the greater the interocular differences, the poorer the driving performance. It is pertinent to understand the negative impact on driving performance that could be implied by increased interocular differences, such as when waiting for a cataract operation on the second eye. Various studies have investigated the effect of cataracts on driving performance. Hwang et al.^77^ found that with both simulated (0.8 Bangerter foil) and real mild cataracts, a substantial negative effect of oncoming headlight glare was measurable when detecting crossing and walking pedestrians, reducing driving performance. In addition, Meuleners et al.^27^ highlighted the negative effect on driving performance (increased crashes) in patients between their first- and second-eye cataract surgery, which caused more intraocular scattering in one eye than in the other, inducing interocular differences. As specified, we also investigated the relationship between driving performance and interocular differences by means of filters that simulate various degrees of cataract, including after moderate alcohol consumption.

This study has certain limitations that should be taken into account. Firstly, the experimental task in the driving simulator does not fully reflect real-world driving behavior, as the subjects are not able to interpret risks in the same way. Nonetheless, the driving simulator is a well-established tool that is efficient, valid, and safe for assessing driving performance under different conditions, including those measured in this study, i.e., filters and alcohol^62,78,79^. Secondly, the study did not include patients with early or mild cataracts to compare these with the filters used to simulate this ocular pathology. Future research could address this limitation by including patients with cataracts at different stages to explore and compare the effects of different degrees of visual impairment on driving performance.

## Conclusion

This study provides a comprehensive framework for understanding the relationships between binocular visual function, interocular differences, and driving performance. Specifically, driving performance is significantly impaired under alcohol consumption and filter conditions (inducing a deterioration in visual performance) Furthermore, the interocular differences due to intraocular scattering (OSI) and straylight (log(s)) are directly related to a reduction in both overall visual and driving performance. In practice, our findings contribute to the understanding of the importance of limiting interocular differences, which can be common among presbyopes corrected using the monovision technique, as well as in cases of cataract surgery on one eye. These interocular differences can have an adverse impact on driving performance and road safety, especially when combined with moderate alcohol consumption.

## Data Availability

The datasets used and analyzed during the current study available from the corresponding author on reasonable request.
